# The multidimensional social ecological needs and service utilization patterns of youth using non-medical prescription opioids: an observational study of routinely collected data in a provincial integrated youth services initiative

**DOI:** 10.1186/s13011-025-00682-6

**Published:** 2025-11-26

**Authors:** Kirsten Marchand, Guiping Liu, Katherine G. Hastings, Heather Palis, Steve Mathias, Jason M. Sutherland, Skye Barbic

**Affiliations:** 1 Department of Occupational Science and Occupational Therapy, Faculty of Medicine, University of British Columbia, 317-2194 Health Sciences Mall, Vancouver, BC V6T 1Z3 Canada; 2 Foundry, 915-1045 Howe Street, Vancouver, BC V6Z 2A9 Canada; 3 Centre for Advancing Health Outcomes, 588-1081 Burrard Street, Vancouver, BC V6Z 1Y6 Canada; 4 Department of Psychiatry, Institute of Mental Health, University of British Columbia, 2255 Wesbrook Mall, Vancouver, BC V6T 2A1 Canada; 5 Centre for Health Services and Policy Research, East Mall, Vancouver, BC 201- 2206, V6T 1Z3 Canada; 6 School of Population and Public Health, University of British Columbia, 2206 East Mall, Vancouver, BC V6T 1Z3 Canada; 7 BC Centre for Disease Control, 655 West 12th Avenue, Vancouver, BC V5Z 4R4 Canada

**Keywords:** Adolescents, Young adults, Youth, Substance use disorder, Non-medical prescription opioid use, Early intervention, Treatment, Integrated care, Integrated youth services

## Abstract

**Background:**

The present study aimed to identify opportunities for earlier intervention of non-medical prescription opioid use (NMPOU) among youth (aged 12–24) by characterizing the multidimensional social ecological needs of youth reporting NMPOU and their service utilization patterns in an expanding integrated youth services network.

**Methods:**

The sample (*n* = 6181) included youth who accessed a novel integrated youth services (IYS) network in British Columbia, Canada, which delivers five core service streams for youth health and well-being through coordinated services. Analyses were conducted on routinely collected data drawn from youths’ self-reported demographic and health measures and service provider-reported service utilization data. Multivariable logistic regression identified factors associated with past 30-day NMPOU and multivariable Poisson regression was used to compare service utilization outcomes between youth with and without NMPOU.

**Results:**

A total of 248 (4%) youth reported past 30-day NMPOU. Multidimensional factors independently associated with NMPOU included poor self-rated physical health compared to excellent/very good physical health (adjusted odds ratio (aOR) = 2.51, 95% Confidence Interval (95% CI) = 1.39, 4.56), high likelihood of externalizing mental health disorders compared to low likelihood (aOR = 1.72, 95% CI = 1.03, 2.86), past 30-day illicit polydrug use compared to none (aOR = 10.00, 95% CI = 5.89, 16.99), and past 3-month exposure to violence compared to none (aOR = 2.18, 95% CI = 1.62, 2.92). Rates of service utilization were similar between youth with and without NMPOU (adjusted relative rate = 1.02, 95% CI = 0.95, 1.32).

**Conclusions:**

These findings indicate that youth with NMPOU present to IYS with several individual, interpersonal, and community-related social ecological needs. Integrated care models may be beneficial to address these multidimensional needs and reduce barriers to service utilization, thereby providing opportunity for earlier intervention of NMPOU among youth. Future research should examine the extent to which IYS reduce the incidence of NMPOU and improve opioid-related health and social outcomes among youth.

## Background

Non-medical prescription opioid use (NMPOU) among youth (typically ages 12–24 years), defined as intentional use of prescription opioids without a prescription or not as prescribed [1], is a critical concern in the context of the ongoing opioid crisis in Canada [2]. The dramatic rise in the number of overdose events has been predominantly driven by illicitly manufactured synthetic opioids, including fentanyl and its analogues over the past decade [3], however, NMPOU among youth remains worrisome for several reasons. NMPOU typically begins and peaks during the adolescent (ages 12–17 years) and young adult (ages 18–24 years) developmental periods [4–8]. NMPOU is also more prevalent among youth than illicit heroin or fentanyl use, with past year prevalence estimates of NMPOU ranging from 4.8–7.5% and 7.6–13.2% among adolescents and young adults, respectively [9–11]. Additionally, a high proportion of youth engage in NMPOU alongside other substances [12] and transition to riskier patterns of opioid use (e.g., injection drug use, unregulated opioid use) [4, 7, 13], which may increase their exposure to illicit fentanyl through counterfeit tablets that resemble prescription drugs or through poly-opioid use.

The evolving complexity of the overdose crisis has revealed the need for broader public health approaches that can effectively respond to the social determinants of opioid use among youth, as early as possible [3, 14–16]. Socio ecological theory [17] can inform these efforts by identifying which social determinants influence youths’ opioid use initiation and opioid use disorder (OUD) [14, 18, 19]. Social ecological frameworks, such as the frequently cited Jalali et al. social ecological framework of the overdose crisis [18], synthesize these environmental and social influences of opioid use at intersecting levels. The individual level of the framework considers the role of individuals’ socio-demographic, biological, health, and psychosocial characteristics on opioid use. The interpersonal level considers how family and peer relationships and experiences shape individuals’ exposure and initiation of opioid use. The communal level considers the influences of factors such as geography, treatment accessibility, and community norms on opioid use. Finally, the societal level of the framework discusses how broader social and health policies affect influences of opioid use, such as government regulations, economic conditions, and drug supply and demand [14, 18].

Understanding which social ecological factors influence NMPOU among youth is therefore valuable to guiding research, practices, and policies that can promote public health responses to youths’ shifting patterns of opioid use, including NMPOU and polydrug use [14, 15]. Past research has reported that biological factors, such as changes in the brain’s reward system, influence risk-taking and sensation-seeking behaviours during the adolescent period [20]. Adverse childhood experiences [9], poor coping and emotion regulation [9, 21, 22], education attainment [23, 24], mental and physical illnesses [9, 25], other substance use [9, 11, 12, 22, 26], and limited awareness of drug-related harms [27] are among the commonly reported social and environmental influences on NMPOU exposure and initiation.

Multidisciplinary and integrated care models, such as those recommended for adults with OUD [28], may promote earlier response to the social ecological determinants of NMPOU among youth through their delivery of comprehensive services. Integrated care models address medical, mental, behavioural, and social health conditions within the same program or treatment system and have shown positive effects on health outcomes compared to usual care [29, 30]. While there are a range of integrated care models [30], integrated youth services (IYS) are one novel and timely delivery system innovation that is specific to the developmental needs of youth. IYS aim to reduce barriers to services for mental and substance use health so that youth can get the services they need as early as possible [29, 31–34]. IYS typically co-locate and coordinate health and social services in low-barrier and youth-centred environments [29, 31–34], which presents a promising opportunity for routine delivery of substance use screening, assessment, and intervention as youth present to services for other health concerns.

To our knowledge, no studies have examined the multidimensional social ecological needs and service utilization patterns of youth with NMPOU in integrated care settings, including IYS. Therefore, the present study aimed to identify opportunities for earlier intervention of NMPOU among youth by characterizing their multidimensional needs and service utilization patterns in an expanding IYS network. The specific objectives were to: [1] identify the multidimensional social ecological factors that relate to NMPOU among youth accessing an expanding network of community-based IYS centres; and [2] compare patterns of service utilization between youth reporting NMPOU and youth not reporting NMPOU in this network of IYS centres, considering service type at first visit and the number of IYS visits. Research in this area is timely given the need to expand developmentally-appropriate and youth-centred responses to the ongoing toxic drug crisis and since IYS are proliferating in Canada and other countries [31, 34]. Where IYS are not yet available, this study informs further research and interventions to promote earlier identification of NMPOU and policies to reduce the harms of NMPOU among youth.

## Method

### Study design and setting

We conducted a retrospective observational study of routinely collected health data drawn from a sample of youth ages 12–24 accessing health or social services at a provincial network of IYS in British Columbia (BC, Canada), between May 1, 2018, and January 31, 2022. The province of BC has over five million residents and declared an overdose public health emergency in 2016 [35]. This BC IYS initiative is branded “Foundry” and is one of the largest IYS networks in Canada [34]. Foundry’s IYS encompass five service streams: mental health, substance use health, physical and sexual health, social services, and peer support. Services are coordinated around youths’ self-reported goals and needs and delivered by multi-disciplinary care teams (e.g., peer support workers, social workers, nurses, physicians) in youth-centred environments, free of charge, without referral, and confidentially [34, 36]. The study followed the REporting of studies Conducted using Observational Routinely collected health Data (RECORD) statement [37] and received research ethics approval by the University of British Columbia/Providence Health Care Research Ethics Board (H22-00522).

### Data sources and sample

Data sources for this study were drawn from individually linked data derived from a youth-reported demographic and health survey and a service provider-reported end of visit form. The demographic and health survey was voluntarily collected for research and evaluation purposes and not required in order to receive services since youth could opt-out of the surveys without it impacting their care [38]. This survey was electronically collected from youth on tablets in the centre waiting room at a single time point – a youths’ initial visit to Foundry – to gather a baseline estimate of youths’ demographic and health status. The demographic and health survey data were individually linked to youths’ service utilization data, which were routinely recorded by service providers at the end of every IYS visit for each youth in an electronic end of visit form [39]. The end of visit form captured data on the date and type(s) of services delivered by service providers during each IYS visit. Possible services types included: [1] physical and sexual health (e.g., birth control, eating/nutrition, trans-care) [2], mental health/substance use health (e.g., mental health counseling, psychosocial rehabilitation, overdose prevention) [3], social support (e.g., housing support, employment information) [4], youth peer support (e.g., centre orientation, non-clinical emotional support, advocacy) [5], walk-in counseling (a low barrier, same-day service for mental health/substance use) [6], and service navigation/group-based services (e.g., referrals, life-skills groups; service types were combined in the present analysis due to their relative low frequency). As an IYS model, youth could engage in more than one service type on a given visit date.

Data from the linked demographic and health survey and end of visit form were de-identified, stored, and analyzed in a centralized secure research environment. To be included in the analytic sample, youth had to have completed the comprehensive set of the demographic and health surveys. The analytic sample comprised a non-random sample of 6,181 unique youth, reflecting 20% of the total number of youth who accessed Foundry’s services during the study period (*n* = 31,025).

### Measurement and variables

#### Objective 1 – Identifying the multidimensional social ecological factors that relate to baseline NMPOU

The outcome of interest was baseline NMPOU in the past 30 days, dichotomously defined as youth who self-reported NMPOU in the 30 days prior to survey completion at initial intake. The comparison group included youth who did not report past 30-day NMPOU. Multidimensional social ecological factors were drawn from the comprehensive youth-reported baseline demographic and health survey. This comprehensive survey uses a combination of validated instruments (e.g., Global Appraisal of Individual Needs – Short Screener [40, 41]) and purpose-built questions. Variables of interest were broadly informed by Jalali et al.’s social ecological framework of the opioid crisis [18], which defines major risk factors for opioid misuse at individual, interpersonal, community, and societal levels [18]. As a secondary analysis, variable selection was limited to factors measured at the individual (e.g., age, gender identity, ethnicity/race, education/employment, physical and mental health, substance use), interpersonal (e.g., family relationships, exposure to violence), and community levels (e.g., housing security, community services) of this framework.

#### Objective 2 – Comparing service utilization patterns between youth with and without NMPOU

Service utilization outcomes of interest included service type (as defined in Sect. “Data sources and sample”) at first visit and total count of IYS visits during the study period [39]. A “service visit” was counted when a service provider registered a unique service date and service type in the end of visit form. These outcomes were compared between youth reporting past 30-day NMPOU and youth not reporting past 30-day NMPOU at initial intake.

### Analysis

#### Statistical methods

Descriptive statistics summarized youths’ characteristics and multidimensional social ecological needs. For objective 1, we conducted univariate and multivariable logistic regression analyses to estimate the association between all selected multidimensional social ecological factors (Table 2) and NMPOU. Prior to running the multivariable model, we suspected that several pairs of variables might be highly correlated (e.g., feeling safe in current living situation and recent exposure to violence), and if put both in a model, could result in collinearity. We used Crammer’s V, a chi-square based measure that determines if two categorical variables were strongly correlated, to confirm our hypotheses regarding the correlation between pairs of conceptually similar variables. If Cramer’s V >0.5, collinearity was confirmed [42], and we then subjectively selected the variable from the pair that best served our study purpose. This process helped us to prepare a group of candidate variables to be included in the multivariable model. Model selection was guided by a lower Akaike information criterion (AIC) and C-score >0.8 and all variables in the final model had a Variance Inflation Factor (VIF) < 6.

For objective 2, descriptive statistics were used to summarize the service type at first visit and total number of service visits during the study period. These statistics were summarized descriptively by NMPOU groups and by whether youth had only a single visit or multiple service visits during the study period. We further conducted multivariable Poisson regression analyses among the sub-sample of youth who had multiple service visits to model the rate of service visits, accounting for differences in youths’ follow-up time. The main effect of interest was baseline past 30-day NMPOU vs. no baseline past 30-day NMPOU. Selection of confounders (age, gender, race/ethnicity, self-rated mental and physical health, and service type at first visit) was informed by results from the multivariable logistic regression and included variables that were deemed to be meaningfully (conceptually or statistically) associated with past 30-day NMPOU and service utilization. For the multivariable Poisson model, we tested for overdispersion by observing if the variance was larger than mean and estimating the Pearson and deviance statistics, which exceeded 1. We therefore adjusted for overdispersion by adding a scaling factor to the final Poisson regression model. Models were not zero inflated since youth visiting IYS usually received at least one service type. All analyses were conducted using SAS^®^ version 9.4.

#### Bias

Item non-response was low on key demographic factors overall (< 7%). However, item non-response was not consistent across NMPOU groups, and assumed to be missing not at random (MNAR) [43]. To prevent loss of statistical power and possible biased estimates due to missing data in the multivariable regression models, we used the missing indicator method [44]. This statistical approach is suitable when data are MNAR and retains all participants in the multivariable analyses by adding an extra indicator representing whether the value for that variable was missing (1/0).

## Results

A total of 248 (4%) youth in the analytic sample reported past 30-day NMPOU. As shown in Table 1, the demographic characteristics of youth with baseline NMPOU in the past 30 days were similar to those of the overall sample with respect to age and self-reported gender and race/ethnicity. Approximately 20% of youth reporting NMPOU were not currently engaged in education/school or employment and 8.9% reported living in insecure housing situations. Frequent reasons for seeking IYS services among youth with NMPOU included their feelings (55.2%), alcohol/drug use (12.5%) and other concerns (12.1%). As shown in Table 2, the proportion of youth reporting other substance use was higher among youth with NMPOU compared to youth without NMPOU, particularly for regular daily marijuana (46.4% vs. 28.2%), regular daily tobacco (52.4% vs. 26.3%), and past 30-day cocaine (25.4% vs. 4.7%) or hallucinogen use (27.0% vs. 6.3%).

**Table 1:** Descriptive characteristics of study sample at initial visit to integrated youth services initiative (*n* = 6181)

	Past 30-day non-medical prescription opioid use		
	Yes (*N* = 248)	No (*N* = 5933)	Total (*N* = 6181)
	*n* (%)	*n* (%)	*n* (%)
**Age Group**			
12–18	110 (44.4)	2970 (50.1)	3080 (49.8)
19–24	136 (54.8)	2903 (48.9)	3039 (49.2)
Missing	< 5	60 (1.0)	< 65 (1.0)
**Gender Identity** ^**a**^			
Female	149 (60.1)	3395 (57.2)	3544 (57.3)
Male	80 (32.3)	1949 (32.9)	2029 (32.8)
Gender diverse/other	19 (7.7)	589 (9.9)	608 (9.8)
**Race/Ethnicity** ^**b**^			
White	163 (65.7)	3773 (63.6)	3936 (63.7)
Indigenous (First Nations, Métis, Inuit)	35 (14.1)	638 (10.8)	673 (10.9)
Visible minority	36 (14.5)	1295 (21.8)	1331 (21.5)
Missing	14 (5.6)	227 (3.8)	241 (3.9)
**In Education and/or Employment**			
In education and/or employment	189 (76.2)	4704 (79.3)	4893 (79.2)
In neither	51 (20.6)	870 (14.7)	921 (14.9)
Missing	8 (3.2)	359 (6.1)	367 (5.9)
**Current Housing Situation** ^**c**^			
Secure	211 (85.1)	5201 (87.7)	5412 (87.6)
Group home	< 5	67 (1.1)	< 71
Insecure	22 (8.9)	257 (4.3)	279 (4.5)
Other	9 (3.6)	135 (2.3)	144 (2.3)
Missing	< 5	273 (4.6)	< 280
**Today**,** I Am Here to Discuss**:			
Alcohol/drugs	31 (12.5)	184 (3.1)	215 (3.5)
Eating/nutrition	< 5	84 (1.4)	86 (1.4)
Family	< 5	145 (2.4)	147 (2.4)
Finances	< 5	26 (0.4)	28 (0.5)
Housing	5 (2.0)	54 (0.9)	59 (1.0)
My feelings (stress, anxiety, depression)	137 (55.2)	3918 (66.0)	4055 (65.6)
Physical health	21 (8.5)	400 (6.7)	421 (6.8)
Relationships	5 (2.0)	111 (1.9)	116 (1.9)
School/work	3 (1.2)	70 (1.2)	73 (1.2)
Sexual health	8 (3.2)	311 (5.2)	319 (5.2)
Other ^d^	30 (12.1)	576 (9.7)	606 (9.8)
Missing	< 5	54 (0.9)	56 (0.9)

**Abbreviations**: NMPOU = non-medical prescription opioid use

**Notes**: (a) Survey asks youth to self-report how they identify, and response options included male, female, and trans female, trans male, non-binary, agender, two-spirit, other (collapsed to “Gender diverse”)

(b) Survey asks youth to self-report their race/ethnicity. Following Statistics Canada, a “Visible minority” category collapsed the following response options: Chinese, Filipino, Japanese, Korean, Latin American, South Asian, Southeast Asian, West Asian, Black, and/or Arab

(c) Survey asks youth to self-report their current housing situation. Variables were collapsed into the following categories: “Secure housing” includes response options for “In a house” and “In an apartment”; “Insecure housing” includes “In a homeless shelter”, “On the street”, “In a single room occupancy”, or “Couch surfing”

(d) Open text examples included all of the above, birth control, attention-deficit hyperactivity disorder (ADHD), gender-affirming care, etc

**Table 2:** Substance use characteristics of study sample at initial visit to integrated youth services initiative (*n* = 6181)

	Past 30-day non-medical prescription opioid use		
	Yes (*N* = 248)	No (*N* = 5933)	Total (*N* = 6181)
	*n* (%)	*n* (%)	*n* (%)
**Lifetime Alcohol Use**			
Never tried	11 (4.4)	658 (11.1)	669 (10.8)
Former user	17 (6.9)	433 (7.3)	450 (7.3)
Regular daily	46 (18.5)	432 (7.3)	478 (7.7)
Social user	141 (56.9)	3011 (50.8)	3152 (51.0)
Tried a few times	29 (11.7)	886 (14.9)	915 (14.8)
Missing	< 5	513 (8.6)	517 (8.4)
**Lifetime Marijuana Use**			
Never tried	27 (10.9)	1272 (21.4)	1299 (21.0)
Former user	30 (12.1)	581 (9.8)	611 (9.9)
Regular daily	115 (46.4)	1672 (28.2)	1787 (28.9)
Social user	43 (17.3)	1087 (18.3)	1130 (18.3)
Tried a few times	26 (10.5)	810 (13.7)	836 (13.5)
Missing	7 (2.8)	511 (8.6)	518 (8.4)
**Lifetime Tobacco Use**			
Never tried	40 (16.1)	1694 (28.6)	1734 (28.1)
Former user	12 (4.8)	508 (8.6)	520 (8.4)
Regular daily	130 (52.4)	1559 (26.3)	1689 (27.3)
Social user	34 (13.7)	688 (11.6)	722 (11.7)
Tried a few times	25 (10.1)	924 (15.6)	949 (15.4)
Missing	7 (2.8)	560 (9.4)	567 (9.2)
**Past 30-day Cocaine or Crack Cocaine Use**			
Yes	63 (25.4)	276 (4.7)	339 (5.5)
No	185 (74.6)	4818 (81.2)	5003 (80.9)
Missing	0 (0.0)	839 (14.1)	839 (13.6)
**Past 30-day Amphetamine Use**			
Yes	28 (11.3)	89 (1.5)	117 (1.9)
No	220 (88.7)	5005 (84.4)	5225 (84.5)
Missing	0 (0.0)	839 (14.1)	839 (13.6)
**Past 30-day Hallucinogen Use**			
Yes	67 (27.0)	373 (6.3)	440 (7.1)
No	181 (73.0)	4721 (79.6)	4902 (79.3)
Missing	0 (0.0)	839 (14.1)	839 (13.6)
**Past 30-day MDMA Use**			
Yes	48 (19.4)	173 (2.9)	221 (3.6)
No	200 (80.6)	4921 (82.9)	5121 (82.9)
Missing	0 (0.0)	839 (14.1)	839 (13.6)
**Past 30-day Illicit Polydrug Use** ^a^			
Yes	32 (12.9)	48 (0.8)	80 (1.3)
No	216 (87.1)	5885 (99.2)	6101 (98.7)

**Abbreviations**: NMPOU = non-medical prescription opioid use; MDMA = 3,4-Methylenedioxymethamphetamine

**Notes**: (a) Includes any use of illicit heroin/fentanyl, cocaine, amphetamine, hallucinogen, or MDMA in past 30 days

Table 3 presents the unadjusted and adjusted odds ratios (aOR) and 95% confidence intervals (95% CI) for the association between multidimensional social ecological needs and past 30-day NMPOU. The adjusted odds of past 30-day NMPOU were significantly lower among youth reporting visible minority race/ethnicity compared to youth reporting White race/ethnicity (aOR = 0.64, 95% CI = 0.44–0.94). Past 30-day NMPOU was significantly higher among youth who reported poor self-rated physical health compared to excellent/very good (aOR = 2.51, 95% CI = 1.39–4.56), had a high likelihood of externalizing mental disorders compared to low likelihood (aOR = 1.72, 95% CI = 1.03–2.86), reported past 30-day illicit polydrug use compared to none (aOR = 10.0, 95% CI = 5.89–16.99), and who had exposure to violence in the past three months compared to none (aOR = 2.18, 95% CI = 1.62–2.92). Of note, exposure to violence was found to be collinear with the community-level factors “current housing situation” and “not feeling safe where I live”. These factors were significantly associated with past 30-day NMPOU in the univariate regression analyses and removed from the multivariable regression model.

**Table 3:** Univariate and multivariable logistic regression of social ecological factors associated with past 30-day Non-medical prescription opioid use (*n* = 6181)

	Unadjusted OR (95% CI)	Adjusted OR (95% CI)
**Individual Factors:**		
**Age Group (Ref. 12–18)**		
19–24	1.27 (0.98, 1.64)	**1.35 (1.00**,** 1.82)**
**Gender Identity (Ref. Male)** ^**a**^		
Female	1.07 (0.81, 1.41)	1.25 (0.92, 1.69)
Gender diverse/other	0.79 (0.47, 1.31)	0.70 (0.41, 1.21)
**Race/Ethnicity (Ref. White)** ^**b**^		
Indigenous (First Nations, Métis, Inuit)	1.17 (0.87, 1.85)	1.01 (0.67, 1.52)
Visible minority	**0.64 (0.45**,** 0.93)**	**0.64 (0.44**,** 0.94)**
**In Education and/or Employment (Ref.)**		
In neither	**1.46 (1.06**,** 2.00)**	0.82 (0.56, 1.20)
**Self-rated Physical Health (Ref. Excellent/Very good)**		
Good	0.96 (0.57, 1.61)	0.90 (0.51, 1.57)
Fair	1.79 (1.09, 2.92)	1.35 (0.78, 2.34)
Poor	**4.99 (3.00**,** 8.29)**	**2.51 (1.39**,** 4.56)**
**Diagnosed Health Conditions (Ref. None)**		
Learning disabilities	1.50 (0.81, 2.77)	1.20 (0.63, 2.28)
Brain injury	1.81 (0.78, 4.23)	1.48 (0.61, 3.63)
ADD or ADHD	1.07 (0.72, 1.61)	0.85 (0.56, 1.29)
Cognitive problems	2.14 (0.97, 4.72)	1.29 (0.55, 3.04)
Two of these conditions	**2.00 (1.37**,** 2.92)**	1.31 (0.87, 1.98)
Three or more of these conditions	**2.25 (1.44**,** 3.50)**	1.14 (0.69, 1.87)
**Likelihood of Internalizing Mental Disorders (Ref. Low likelihood)** ^**c**^		
Moderate likelihood	0.82 (0.38, 1.81)	0.68 (0.30, 1.56)
High likelihood	1.86 (0.95, 3.66)	0.88 (0.42, 1.87)
**Likelihood of Externalizing Mental Disorders (Ref. Low likelihood)** ^**c**^		
Moderate likelihood	1.30 (0.83, 2.02)	1.08 (0.67, 1.76)
High likelihood	**2.77 (1.78**,** 4.30)**	**1.72 (1.03**,** 2.86)**
**Prior 30-day Illicit Polydrug Use (Ref. No)** ^d^		
Prior 30-day illicit polydrug use	**18.16 (11.38**,** 28.99)**	**10.00 (5.89**,** 16.99)**
**Taking Care of My Health Is (Ref. Very easy)**		
Easy	0.69 (0.30, 1.56)	0.54 (0.23, 1.30)
Difficult	1.24 (0.57, 2.69)	0.70 (0.29, 1.66)
Very difficult	**2.89 (1.32**,** 6.30)**	0.96 (0.39, 2.36)
**Interpersonal Factors:**		
**Family Support (Ref. Yes**,** about most things)**		
Sometimes, depending on the problem	**1.49 (1.08**,** 2.05)**	1.30 (0.92, 1.82)
No	**1.73 (1.16**,** 2.57)**	1.21 (0.79, 1.85)
**Seen or Experienced Violence in Past 3 months (Ref. No)**		
Seen or experienced violence in past 3 months	**3.10 (2.37**,** 4.06)**	**2.18 (1.62**,** 2.92)**
**Community Factors:**		
**Current Housing Situation (Ref. Secure)** ^**e, f**^		
Insecure	**2.11 (1.34**,** 3.33)**	**–**
Group home	1.10 (0.34, 3.54)	**–**
Other	1.64 (0.82, 3.27)	**–**
**Feeling Safe Where I Live (Ref. Yes)** ^**f**^		
Not feeling safe where I live	**2.28 (1.63**,** 3.17)**	**–**

**Abbreviations and Notes**: ADD = attention deficit disorder; ADHD = attention-deficit hyperactivity disorder OR = odds ratio; 95% CI = 95% confidence interval; Ref. = reference category (i.e., OR = 1.0); bolded ORs indicate statistical significance at *p* < 0.05

(a) Survey asks youth to self-report how they identify, and response options include male, female, and trans female, trans male, non-binary, agender, two-spirit, other (collapsed to “Gender diverse”)

(b) Survey asks youth to self-report their race/ethnicity. Following Statistics Canada, a “Visible minority” category collapsed the following response options: Chinese, Filipino, Japanese, Korean, Latin American, South Asian, Southeast Asian, West Asian, Black, and/or Arab

(c) Derived from the substance use subscale of the Global Appraisal of Individual Needs – Short Screener (GAIN-SS). Sub-scale scores range from 0 to 5 for past month. According to scale norms, 0 symptoms is considered low likelihood of substance use service need, 1–2 symptoms is moderate likelihood of substance use service need, and 3 + symptoms is considered high likelihood of substance use service need

(d) Reporting > 1 illicit drug type in past 30 days, including heroin/fentanyl, cocaine, amphetamine, hallucinogen, or MDMA use

(e) Survey asks youth to self-report their current housing situation. Variables were collapsed into the following categories: “Secure housing” includes response options for “In a house” and “In an apartment”; “Insecure housing” includes “In a homeless shelter”, “On the street”, “In a single room occupancy”, or “Couch surfing”

(f) Variable was not included in multivariable logistic regression analysis as it was collinear with the variable “Seen or experienced violence in past 3 months”

Regarding service utilization patterns, the proportion of youth reporting NMPOU was similar across samples of youth with one IYS visit (3.3%) and multiple IYS visits (4.3%) **(**Table 4**)**. The distribution of service type at first visit slightly differed by whether youth had one visit or multiple visits. A higher proportion of youth with one IYS visit accessed mental health/substance use services (38.6% vs. 29.6%) or walk-in services (36.0% vs. 29.7%) on their first visit compared to youth with two or more visits. This pattern was consistent among youth with and without NMPOU. Among the sample of youth who had more than one IYS visit during the study period (*n* = 4314), results from the multivariable Poisson regression model suggested that youth with and without NMPOU had a similar relative rate (RR) of IYS visits (RR = 1.02; 95% CI = 0.95–1.32), after controlling for first visit service type(s) and confounders (Table 5).

**Table 4:** Service utilization patterns for youth with and without past 30-day Non-medical prescription opioid use (*n* = 6181)

	Among Youth Who Had One IYS Visit Only (*N* = 1867, 30.2%)			Among Youth Who Had Multiple IYS visits (*N* = 4314, 69.8%)		
	Yes NMPOU (*N* = 62, 3.3%)	No NMPOU (*N* = 1805, 96.7%)	Total (*N* = 1867, 100%)	Yes NMPOU (*N* = 186, 4.3%)	No NMPOU (*N* = 4128, 95.7%)	Total (*N* = 4314, 100%)
**Service Type(s) at First IYS Visit, n (%)**						
Only physical and sexual health	6 (9.7)	174 (9.6)	180 (9.6)	32 (17.2)	713 (17.3)	745 (17.3)
Only mental health/substance use (MHSU)	20 (32.3)	700 (38.8)	720 (38.6)	55 (29.6)	1222 (29.6)	1277 (29.6)
Only social support	< 5	11 (0.6)	13 (0.7)	6 (3.2)	60 (1.5)	66 (1.5)
Only youth peer support	< 5	20 (1.1)	21 (1.1)	< 5	40 (1.0)	41 (1.0)
Only walk-in counseling	23 (37.1)	650 (36.0)	673 (36.0)	48 (25.8)	1232 (29.8)	1280 (29.7)
Only navigation/groups ^a^	< 5	30 (1.7)	31 (1.7)	5 (2.7)	137 (3.3)	142 (3.3)
1 + Service type, including MHSU ^b^	5 (8.1)	175 (9.7)	180 (9.6)	31 (16.7)	548 (13.3)	579 (13.4)
1 + Service type, excluding MHSU ^b^	< 5	45 (2.5)	49 (2.6)	8 (4.3)	168 (4.1)	176 (4.1)
**Total Number of Visits Over Time**						
Mean (SD)	–	–	–	17.4 (29.52)	15.0 (25.66)	15.1 (25.84)
Median	–	–	–	7	6	6
Interquartile range	–	–	–	3.0, 17.0	3.0, 16.0	3.0, 16.0

**Abbreviations and Notes**: IYS = integrated youth services; NMPOU = non-medical prescription opioid use; SD = standard deviation; MHSU = mental health and substance use

(a) These service types were collapsed due to small counts

(b) In these IYS visits, multiple service types were recorded on the same date. Given the large number of possible combinations of service types, we differentiated those with multiple service types including MHSU and excluding MHSU, the most frequent service type accessed at the first visit for all groups

**Table 5:** Multivariable Poisson regression of association between Non-medical prescription opioid use and rate of service visits among youth who had multiple service visits (*n* = 4314)

Parameter	Unadjusted RR (95% CI)	Adjusted RR (95% CI)
**Past 30-day NMPOU (Ref. None)**		
Past 30-day NMPOU	1.16 (0.98, 1.36)	1.02 (0.95, 1.32)
**Age Group (Ref. 12–18)**		
19–24	**1.20 (1.12**,** 1.28)**	**1.14 (1.07**,** 1.23)**
**Gender Identity (Ref. Male)** ^a^		
Female	**1.15 (1.06**,** 1.25)**	**1.11 (1.02**,** 1.20)**
Gender diverse/other	**1.68 (1.50**,** 1.87)**	**1.56 (1.40**,** 1.74)**
**Race/ethnicity (Ref. White)** ^b^		
Indigenous (First Nations, Métis, Inuit)	**1.11 (1.00**,** 1.24)**	1.09 (0.98, 1.21)
Visible minority	1.02 (0.94, 1.12)	1.04 (0.96, 1.13)
**Self-rated Physical Health (Ref. Excellent/Very good)**		
Good	**1.34 (1.17**,** 1.52)**	**1.26 (1.11**,** 1.43)**
Fair	**1.50 (1.32**,** 1.71)**	**1.35 (1.19**,** 1.54)**
Poor	**1.49 (1.28**,** 1.74)**	**1.31 (1.12**,** 1.53)**
**Likelihood of Internalizing Mental Disorders (Ref. Low likelihood)** ^c^		
Moderate likelihood	1.20 (0.99, 1.46)	**1.22 (1.01**,** 1.48)**
High likelihood	**1.29 (1.08**,** 1.54)**	**1.31 (1.09**,** 1.57)**
**Likelihood of Externalizing Mental Disorders (Ref. Low likelihood)** ^c^		
Moderate likelihood	1.07 (0.97, 1.18)	1.01 (0.91, 1.12)
High likelihood	0.93 (0.65, 1.37)	0.91 (0.81, 1.02)
**Prior 30-day Illicit Polydrug Use (Ref. No)** ^d^		
Prior 30-day illicit polydrug use	1.20 (0.90, 1.59)	1.07 (0.80, 1.43)
**Service Type(s) at First IYS Visit (Ref. Only walk-in counseling)** ^e^		
Only physical and sexual health	**1.43 (1.29**,** 1.58)**	**1.36 (1.23**,** 1.51)**
Only mental health/substance use (MHSU)	1.05 (0.95, 1.15)	1.01 (0.92, 1.11)
Only social support	**1.72 (1.35**,** 2.19)**	**1.60 (1.26**,** 2.04)**
Only youth peer support	0.69 (0.43, 1.11)	0.68 (0.43, 1.08)
Only navigation/groups ^f^	1.14 (0.93, 1.40)	1.08 (0.88, 1.32)
1 + Service type, including MHSU ^g^	**1.65 (1.48**,** 1.83)**	**1.56 (1.41**,** 1.74)**
1 + Service type, excluding MHSU ^g^	**1.56 (1.32**,** 1.84)**	**1.48 (1.26**,** 1.74)**

**Abbreviations and Notes**: RR = relative rate; 95% CI = 95% confidence interval; NMPOU = non-medical prescription opioid use; MHSU = mental health and substance use; bold indicates statistical significance at *p* < 0.05

(a) Survey asks youth to self-report how they identify, and response options include male, female, and trans female, trans male, non-binary, agender, two-spirit, other (collapsed to “Gender diverse”)

(b) Survey asks youth to self-report their race/ethnicity. Following Statistics Canada, a “Visible minority” category collapsed the following response options: Chinese, Filipino, Japanese, Korean, Latin American, South Asian, Southeast Asian, West Asian, Black, and/or Arab

(c) Derived from the substance use subscale of the Global Appraisal of Individual Needs – Short Screener (GAIN-SS). Sub-scale scores range from 0 to 5 for past month. According to scale norms, 0 symptoms is considered low likelihood of substance use service need, 1–2 symptoms is moderate likelihood of substance use service need, and 3 + symptoms is considered high likelihood of substance use service need

(d) Reporting > 1 illicit drug type in past 30 days, including heroin/fentanyl, cocaine, amphetamine, hallucinogen, or MDMA use

(e) The reference category “Walk-in counseling” was selected for conceptual and statistical reasons; it is a solution-focused, single-session, low-barrier service that is one of the most frequently accessed

(f) These service types were collapsed due to small counts

(g) In these IYS visits, multiple service types were recorded on the same date. Given the large number of possible combinations of service types, we differentiated those with multiple service types including MHSU and excluding MHSU, the most frequent service type accessed at the first visit for all groups

## Discussion

### Multidimensional social ecological factors that relate to baseline NMPOU

In this cohort study of youth seeking services at a novel provincial IYS initiative, we found clinically and statistically significant associations between several social ecological risk factors and NMPOU among youth. As anticipated, the factors most strongly associated with NMPOU included past 30-day illicit polydrug use and recent exposure to violence, which was collinear with the community-level factors current housing situation and not feeling safe in current living situation. As these measures were collected cross-sectionally, our study cannot draw conclusions about the causal pathway between these associations. For instance, we are unable to determine if NMPOU caused polydrug use or recent exposure to violence or if polydrug use or recent exposure to violence caused NMPOU. However, the strength of these independent associations is congruent with broader social ecological [18] and risk environment frameworks for drug use [45, 46]. Such frameworks posit that social and environmental conditions, such as family relationships, peer norms and influences, and neighborhood environments, intersect with structural conditions, such as poverty, racism, and drug criminalization, and increase one’s risk of being exposed to drugs, accessing and initiating drug use, and transitioning to more harmful drug use patterns and outcomes [46, 47]. It is possible that youth in our study transitioned from NMPOU to polydrug use, with this progression influenced by their risk environment. Future explanatory research is needed to understand the age of onset and temporality of these associations given the time-varying nature of youths’ social and environmental contexts and drug use patterns, as these have implications to designing and implementing developmentally appropriate interventions.

### Comprehensive and integrated care models for NMPOU among youth

The strong association between the multidimensional social ecological factors, including polydrug use, and NMPOU indicates an imminent need for a comprehensive and integrated response. Indeed, recent calls to action from youth who use substances and service providers have highlighted the need for low-barrier, youth-dedicated, and youth-led harm reduction and substance use services and spaces, including safer consumption, drug checking, and housing programs that accommodate the intersecting social ecological needs of youth who use drugs [48–50]. These calls to action have also demonstrated youths’ desire for more relatable knowledge of drug-related harms and services to be delivered by a mix of peers and professionals to promote trust and transparency [27, 48, 51, 52]. Therefore, our findings add to the growing body of evidence indicating the need for youth-oriented harm reduction and substance use services, and, in particular, services that can comprehensively respond to intersecting social ecological risks for NMPOU and polydrug use [48, 53, 54].

A comprehensive response to NMPOU and its associated social ecological risks requires a multisectoral approach where the health, education, and social sectors collaborate to align their substance use prevention efforts [14]. Integrated care models, such as IYS, are one such emergent approach, and may therefore present a crucial opportunity to respond to the complex set of health and social needs among youth with NMPOU. In our study, the multidimensional social ecological factors that were associated with NMPOU aligned directly with the service streams available in IYS settings. For instance, the IYS setting presents increased opportunities to address the physical health needs of youth with NMPOU through on site primary health care services. Likewise, trauma from exposure to violence can be responded to through the integrated mental health and social services. Moreover, it is plausible that the co-location of multiple services reduces or eliminates barriers to service engagement that have been previously found to result from stigma and limited knowledge of substance use harms [47, 49, 55–62]. Thus, IYS may also promote earlier identification and care for NMPOU and other substance use by presenting youth with multiple service streams through which they can engage in services.

Several findings from our study support this hypothesis. In particular, the prevalence of NMPOU among youth accessing IYS was higher than that reported in a recent provincial survey of adolescent youth NMPOU prevalence (4% vs. 1%) [63]. Additionally, we found that youth with NMPOU accessed several service streams on their first IYS visit, with mental health/substance use services, walk-in counseling, and physical and sexual health services being the most frequent types of services accessed. Since this pattern was similar for youth with and without NMPOU, it is possible that youth with NMPOU initially sought services for other health and social concerns that were less stigmatize and/or more acute, such as for poor physical health or recent exposure to violence, which were independently associated with NMPOU. We also found minimal differences between youth with and without past 30-day NMPOU in the rate of IYS visits, indicating that youth with NMPOU engaged in services to a similar extent as youth without NMPOU. This is a promising finding considering the pervasive availability and accessibility barriers to services (e.g., location, hours, wait times) that youth with NMPOU and/or polydrug use face in traditional substance use service settings [49, 55–62].

These encouraging findings align with developing research regarding the benefits of integrated care models for adults with OUD [28], such as hub-and-spoke or primary care office-based models. Such models aim to reduce barriers to treatment by ensuring there is “no wrong door” and expanding the settings in which adults can access treatment for OUD [64, 65]. In the hub-and-spoke model, this is achieved by connecting specialized opioid treatment providers (hubs) with multidisciplinary primary, behavioural, and social care teams (spokes) [65]. In primary care office-based models, treatments for opioid use/OUD (e.g., buprenorphine) are integrated into primary care settings [64]. Among adults, emerging evidence suggests that these models reduce barriers to care, improve health and social outcomes, are cost effective, and meet clients’ preferences and expectations [28, 64–67]. As IYS continue to rapidly expand, additional research is needed to strengthen conclusions regarding their benefits for youth with NMPOU, including research on youth and service providers’ perspectives of how IYS reduces barriers to care and longitudinal research on youths’ NMPOU outcomes and IYS experiences.

### Strengths and limitations

This study has several strengths, including linked self-report and service utilization data from a large cohort of youth accessing services across diverse communities in BC. However, our study drew on data collected among youth seeking IYS and lacked a comparison group of youth not exposed to IYS. Therefore, our findings are not likely representative of the general population of youth with NMPOU in BC. Additionally, the self-reported data on NMPOU and other key measures were generated from a sample of youth who voluntarily responded to the comprehensive set of demographic and health measures. This likely underestimates the true prevalence of NMPOU among youth accessing IYS since youth who voluntarily complete the measure of NMPOU may be healthier than youth who did not respond to those surveys. This study design may also minimize differences between youth reporting NMPOU and the comparison group of youth seeking IYS and not reporting NMPOU since the comparison group represents service seeking youth and not a random sample of general population controls. Our analysis of the multidimensional needs of youth with NMPOU was also limited in estimating NMPOU severity and the dynamic nature of relationships between the exposures and NMPOU. In our efforts to control for this, we selected a past 30-day measure of NMPOU instead of the available “lifetime” binary measure (yes/no). Nevertheless, the past 30-day measure of NMPOU likely includes youth with varying patterns of NMPOU (e.g., daily and non-daily use) and may have influenced some of the associations. The noteworthy changes in the magnitude and direction of association for some factors, such as high likelihood of internalizing disorders, in the multivariable logistic regression analysis also suggested the possible presence of residual confounding or mediation [68]. Additionally, to be able to consider other substance use as a multidimensional need of youth with NMPOU, we opted to include these in the case and comparison group definitions. Indeed, as evidenced in the multivariable regression analysis, illicit polydrug use was a strong risk factor for NMPOU, and this may have diminished some of the differences between NMPOU groups. Finally, it is important to note that the data used in our study span the COVID-19 pandemic, which has been shown to have impacted substance use among youth and service utilization patterns [69, 70]. Though the impacts of the pandemic were beyond the scope of the present study, it may be beneficial to replicate this study in the future to determine if the effects of the pandemic on substance use and service utilization have been sustained [71].

## Conclusions

Amid the ongoing drug toxicity crisis, scientists, service providers, and policymakers are urgently called upon to identify effective models of care to curb the incidence of non-medical opioid use and reduce the escalating opioid-related harms among youth. This study provides initial evidence of the potential benefits of integrated care models for engaging youth with NMPOU into services that can respond to their multidimensional care needs. As IYS continue to expand, ongoing research is needed to determine the extent to which IYS reduce the incidence of NMPOU and improve opioid-related health and social outcomes among youth.

## Data Availability

Foundry Data contain sensitive health and personal information, including from minors, and the ethics permission for this study and consent procedures used for collecting data restrict data access only to individuals who meet the criteria for access. Hence, no data are available due to ethical and legal restrictions. Access to data provided by Foundry is subject to approval but can be requested for research projects through Foundry Data Stewards. This research project has undergone a thorough review and approval by Foundry Central Office Data Stewards to ensure that the research methods, data collection procedures, and data management practices complied with established data governance and stewardship policies and procedures. All inferences, opinions, and conclusions drawn in this publication are those of the author(s), and do not necessarily reflect the opinions or policies of Foundry.
